# EGR1 regulates hepatic clock gene amplitude by activating *Per1* transcription

**DOI:** 10.1038/srep15212

**Published:** 2015-10-16

**Authors:** Weiwei Tao, Jing Wu, Qian Zhang, Shan-Shan Lai, Shan Jiang, Chen Jiang, Ying Xu, Bin Xue, Jie Du, Chao-Jun Li

**Affiliations:** 1 MOE Key Laboratory of Model Animals for Disease Study, Model Animal Research Center (MARC) and the School of Medicine, Nanjing University, Nanjing 210093, China; 2 Beijing Anzhen Hospital, Capital Medical University, Beijing, 100029, China

## Abstract

The mammalian clock system is composed of a master clock and peripheral clocks. At the molecular level, the rhythm-generating mechanism is controlled by a molecular clock composed of positive and negative feedback loops. However, the underlying mechanisms for molecular clock regulation that affect circadian clock function remain unclear. Here, we show that *Egr1* (early growth response 1), an early growth response gene, is expressed in mouse liver in a circadian manner. Consistently, *Egr1* is transactivated by the CLOCK/BMAL1 heterodimer through a conserved E-box response element. In hepatocytes, EGR1 regulates the transcription of several core clock genes, including *Bmal1*, *Per1*, *Per2*, *Rev-erbα and Rev-erbβ*, and the rhythm amplitude of their expression is dependent on EGR1’s transcriptional function. Further mechanistic studies indicated that EGR1 binds to the proximal region of the *Per1* promoter to activate its transcription directly. When the peripheral clock is altered by light or feeding behavior transposition in *Egr1*-deficient mice, the expression phase of hepatic clock genes shifts normally, but the amplitude is also altered. Our data reveal a critical role for EGR1 in the regulation of hepatic clock circuitry, which may contribute to the rhythm stability of peripheral clock oscillators.

Most living organisms exhibit behavioral and physiological rhythms, including sleep-wake, rest-activity, blood pressure, hormone secretion and energy metabolism^1^,^2^,^3^,^4^,^5^,^6^. This diurnal oscillation is regulated by circadian clocks, which respond to light and feeding cycles. In mammals, a master clock that generates circadian rhythms is located in the suprachiasmatic nucleus (SCN) and drives the slave oscillators that are distributed in various peripheral tissues using autonomic innervation and behavioral and neuroendocrine signals^6^,^7^,^8^. In addition, behavioral and neuroendocrine signals are innervated by the autonomous nervous system^8^. The SCN synchronizes peripheral oscillators by secreting cyclical neuroendocrine signals or imposing rest-activity rhythms and feeding-fasting cycles^7^,^9^. Feeding behavior regulates the peripheral oscillators through signaling metabolites such as glucose and hormones^7^,^9^.

The central and peripheral clocks are controlled by a common transcriptional circuitry that generates rhythmic patterns of gene expression. The molecular components of the clock oscillator have been elegantly elucidated over the past two decades. At the molecular level, CLOCK and BMAL1 are two basic helix-loop-helix transcription factors that initiate the transcription of target genes that contain E-box *cis*-regulatory enhancer sequences, including *Per* and *Cry*^10^. PER and CRY proteins form heterodimers that translocate back to the nucleus to repress their own expression by repressing CLOCK/BMAL1 complex activity, which forms the critical negative feedback loop within the clock circuitry^11^,^12^. In addition, *Bmal1* transcription is regulated both positively and negatively by the orphan nuclear receptors RORs and REV-ERBs^13^,^14^. This core feedback loop requires approximately 24 h to complete a cycle and constitutes a circadian oscillation of the molecular clock. The proper phases and amplitudes of the endogenous molecular clocks are crucial for the operation of the body’s biological rhythm. However, the significance of amplitude in the function of the circadian pacemaker is not well understood. Previous reports have shown that a robust circadian oscillator might be more resistant to phase perturbation^15^,^16^. The degradation of circadian rhythms in old age is normally accompanied by both loss of amplitude and fragmentation of output rhythms^17^. The reduction in the circadian amplitude may contribute to the instability of circadian rhythms and other homeostatic processes^18^.

*Egr1* is a member of the immediate early response gene family and recognizes a highly conserved GC-rich promoter consensus motif^19^,^20^. *Egr1* can be transiently activated by many cytokines, growth factors, hormones and DNA-damaging agents. In addition, as a transcription factor, EGR1 binds to target sequences and regulates the expression of many genes such as *TNF-α, PTEN* and *SOCS-*1^21–23^. EGR1 expression is highly responsive to nutrition signaling under both fasting and feeding conditions. In the liver, fasting induces EGR1 expression, which promotes hepatic gluconeogenesis by activating *C/EBPα* transcription^24^. EGR1 also activates cholesterol biosynthetic gene expression to promote cholesterol biosynthesis under feeding conditions^25^. Previous reports have also shown that EGR1 can regulate the expression of certain clock genes in cell lines^26^,^27^,^28^. Based on these findings, we hypothesize that EGR1 may play a potential role in the regulation of the hepatic circadian clock in response to feeding/fasting or other signals.

Here, we show that EGR1 is rhythmically expressed in mouse liver and is required for defining some of the circadian expression patterns of several core clock genes in liver. Moreover, we clarify that EGR1 regulates the circadian amplitude of the hepatic clock gene rhythms by activating *Per1* transcription.

## Results

### Egr1 is rhythmically expressed in mouse liver

**Figure 1:**
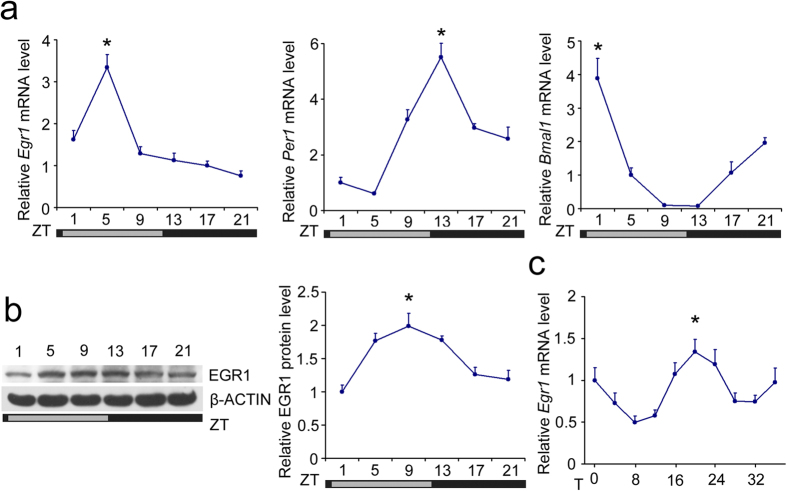
Circadian expression of *Egr1* in mouse liver. (**a**) qRT-PCR analysis of *Egr1*, *Per1* and *Bmal1* mRNA expression in the livers from B6 mice entrained to an LD 12:12 cycle. Data are shown as the mean ± s.d. *P < 0.02 peak versus nadir. (**b**) Protein expression of EGR1 at the indicated time-points. The gels were run under the same experimental conditions. The full-length blots are presented in Supplementary Fig. 5. *P < 0.03 peak versus nadir. (**c**) Time course expression of *Egr1* in mouse AML-12 cells subjected to serum shock. *P < 0.03 peak versus nadir.

Thousands of genes are rhythmically expressed either cell-autonomously or in response to the clockwork of the body. Previous microarray analysis and meta-analysis have shown that *Egr1* is expressed in mouse liver in a circadian manner^29^,^30^,^31^,^32^. At the mRNA level, we also found that *Egr1* expression had a diurnal rhythm that peaked at ZT5 (ZT0 is the onset at hour 0 of the subjective light period), gradually declined thereafter, and reached a nadir at ZT21 in mouse liver (Fig. 1a). The peak of the *Per1* oscillation occurred behind that of *Egr1* but before that of *Bmal1* and other clock genes, implying a potential regulatory relationship between *Egr1* and *Per1* (Fig. 1a, Supplementary Fig. 1a). *Egr1* mRNA was also expressed in a circadian manner in the kidney, skeletal muscle, heart, and epididymal fat; however, the phase differed significantly from the expression pattern observed in the liver (Supplementary Fig. 1b). Immunoblot analysis indicated that EGR1 protein expression was significantly higher at ZT5 to ZT13 and gradually declined to the basal level (Fig. 1b). The rhythmic expression of *Egr1* was also confirmed in a serum shock cell model, in which the peak of *Egr1* mRNA expression occurred approximately 20 hours after serum shock (Fig. 1c).

### Egr1 is regulated under a circadian feedback loop

**Figure 2:**
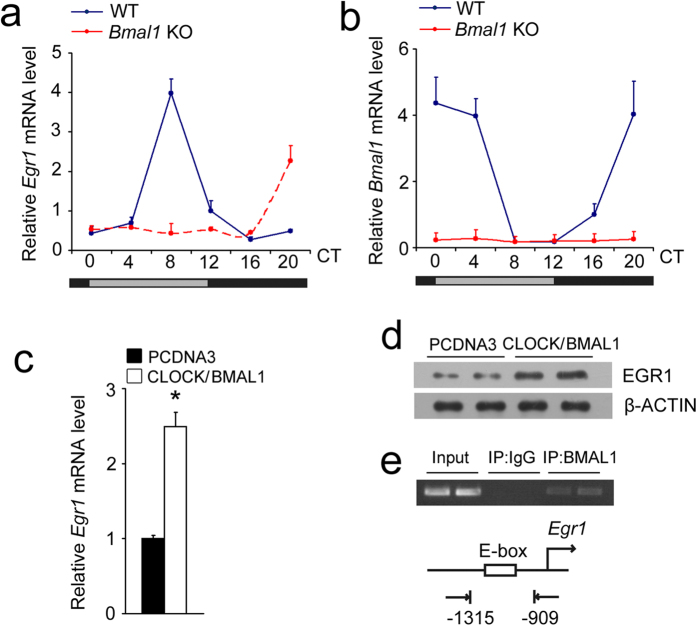
*Egr1* is a clock-controlled gene. (**a**) qRT-PCR analysis of *Egr1* mRNA expression in livers from wild-type and *Bmal1* null mice under constant darkness. (**b**) qRT-PCR analysis of *Bmal1* mRNA expression in livers from wild-type and *Bmal1* null mice under constant darkness. (**c**) qRT-PCR analysis of *Egr1* expression in AML-12 cells after co-transfection with BMAL1 and CLOCK plasmids for 48 h. (**d**) Immunoblot analysis of EGR1 expression in AML-12 cells after co-transfection with BMAL1 and CLOCK plasmids for 48 h. The gels were run under the same experimental conditions. The full-length blots are presented in Supplementary Fig. 6. (**e**) ChIP assays in mouse livers at ZT5. PCR primers amplify a fragment flanking the E box on the *Egr1* promoter. Data are shown as the mean ± s.d. *P < 0.01.

To examine whether the circadian expression of *Egr1* in the liver is regulated by an endogenous circadian clock, we monitored the *Egr1* mRNA levels in the livers of *Bmal1* knockout mice. As shown in Fig. 2a, the phase of the *Egr1* rhythm was delayed for 12 hours, and the amplitude was reduced in the *Bmal1* KO mice under constant darkness. The *Bmal1* expression levels in the livers of *Bmal1* KO mice are shown in Fig. 2b. The overexpression of BMAL1/CLOCK heterodimers in a mouse hepatocyte cell line increased EGR1 expression (Fig. 2c,d). ChIP assays in the mouse liver also revealed that BMAL1 was present near an E box on the proximal *Egr1* promoter at ZT5 (Fig. 2e). These results indicate that *Egr1* is a circadian clock target and is negatively regulated by the circadian feedback loop.

### EGR1 is responsible for the expression of clock genes

**Figure 3:**
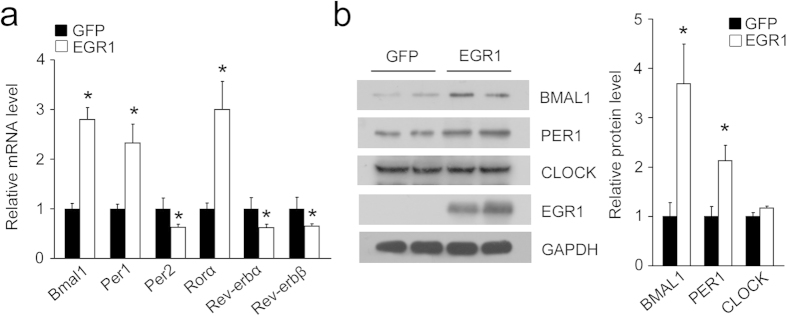
Regulation of clock genes by EGR1. (**a)** qPCR analysis of clock genes in primary hepatocytes infected with GFP or EGR1 adenoviruses for 48 h. *P < 0.04. (**b**) Immunoblots of cell lysates of primary hepatocytes using the indicated antibodies. The gels were run under the same experimental conditions. The full-length blots are presented in Supplementary Fig. 7. Data are shown as the mean ± s.d. *P < 0.05.

To determine whether EGR1 might influence the clock network, we overexpressed EGR1 in primary hepatocytes and in the mouse AML-12 cell line. qPCR analysis indicated that EGR1 was able to induce the expression of *Bmal1*, *Per1* and *Rorα* and to decrease the expression of *Per2*, *Rev-erbα* and *Rev-erbβ* in both cell types (Fig. 3a, Supplementary Fig. 2b). However, other clock genes (*Clock*, *Cry1*, *Cry*2 and *Rorγ*) were not altered, which indicated that EGR1 exerts specific effects on the circadian downstream genes (Supplementary Fig. 2a,b). Consistent with the mRNA expression findings, the BMAL1 and PER1 protein levels were elevated but CLOCK was unaffected upon EGR1 overexpression (Fig. 3b). Our data suggest that EGR1 is expressed autonomously and rhythmically in the liver and regulates the transcription of clock genes.

### EGR1 is responsible for the amplitude but not the phase of the rhythmic clock gene expression in cultured hepatocytes

**Figure 4:**
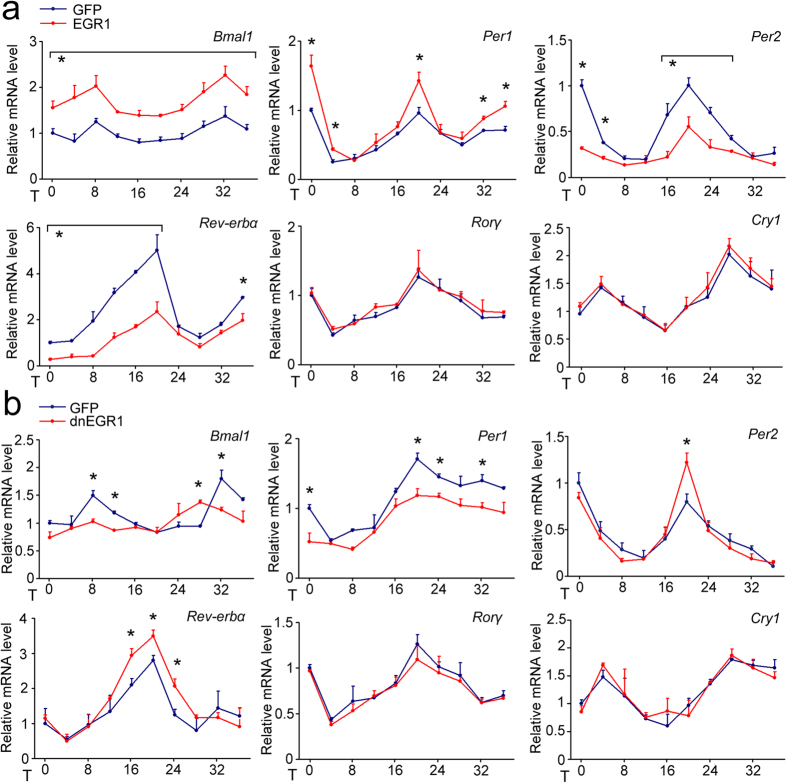
Cell-autonomous role of EGR1 in clock regulation *in vitro*. (**a**) Time course expression of clock genes in mouse AML-12 cells infected with GFP or EGR1 adenoviruses following serum shock. Data are shown as the mean ± s.d. *P < 0.05, EGR1 versus control. (**b**) Time course expression of clock genes in mouse AML-12 cells infected with GFP or dnEGR1 adenoviruses following serum shock. *P < 0.05, dnEGR1 versus control.

Serum shock has been demonstrated to induce rhythmic clock gene expression in cultured cells and thus provides a useful *in vitro* model for studying the clock mechanism^33^. Based on the above results, we further investigated the function of EGR1 in the clock circuit of cultured cells. We overexpressed EGR1 or inhibited the transcriptional activity of EGR1 in mouse AML-12 cells and then exposed these cells to brief serum shock for 2 hours, followed by observation for 36 hours. We found that the phase of the rhythmic clock gene expression was not altered but that the amplitudes of the *Bmal1* and *Per1* rhythms were elevated significantly after EGR1 overexpression, whereas the amplitudes of the *Per2* and *Rev-erbα* rhythms were reduced (Fig. 4a). The inhibition of EGR1 transcriptional activity using dnEGR1 adenovirus reduced the amplitudes of the *Bmal1* and *Per1* rhythms and elevated those of the *Per2* and *Rev-erbα* rhythms (Fig. 4b). Overexpressed EGR1 or transcriptionally repressed EGR1 did not affect the rhythmic expression of *Rorγ* or *Cry1* (Fig. 4a,b). These *in vitro* data suggest that EGR1 dominantly affects the amplitude rather than the phase of rhythmic clock gene expression. Based on the above observations, our results suggest that EGR1 may activate *Per1* transcription. The accumulation of PER1 leads to the repression of *Per2* and *Rev-erbs* by inhibiting BMAL1/CLOCK activity. Repression of REV-ERBs possibly leads to the robust oscillation of *Bmal1*.

### EGR1 is responsible for the amplitude of clock gene rhythms *in vivo*

**Figure 5:**
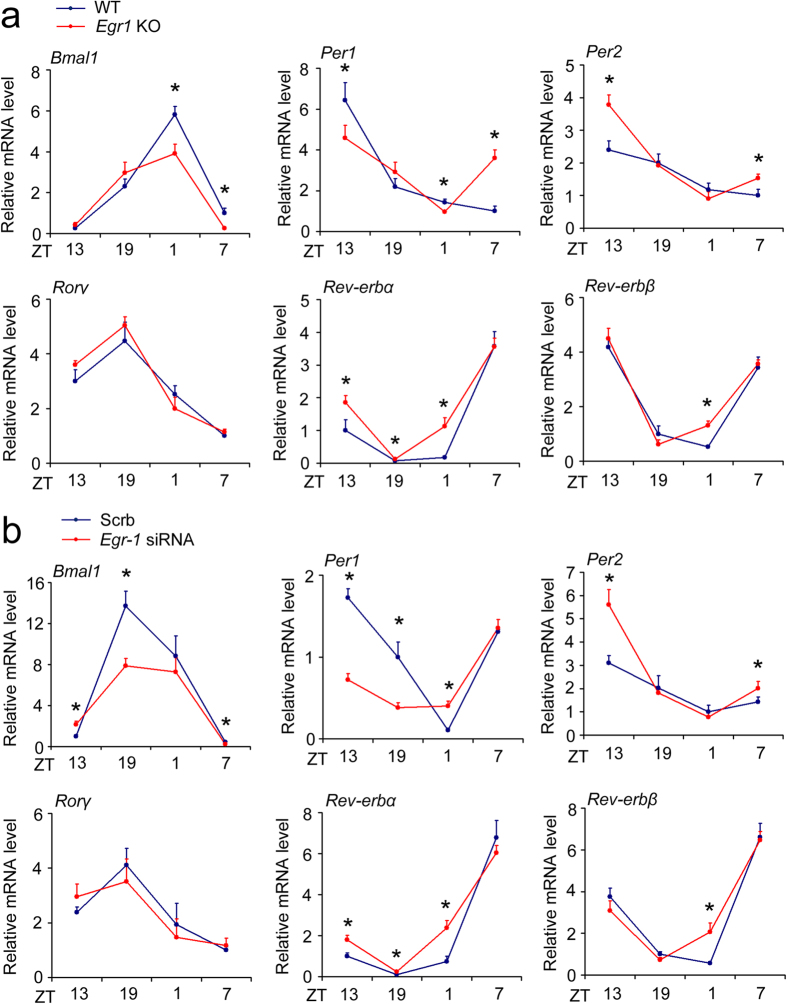
Knockout of *Egr1* or inhibition of hepatic *Egr1* expression disrupted hepatic clock function. (**a**) qRT-PCR analysis of clock gene expression in livers from wild-type and *Egr1* null mice. Data are shown as the mean ± s.d. *P < 0.05, wild-type versus null mice. (**b**) qRT-PCR analysis of clock gene expression in livers from B6 mice injected with Scrb or *Egr1* siRNA through tail vein injections. *P < 0.05, *Egr1* siRNA versus Scrb.

To further confirm the regulation of the amplitude of clock gene rhythms by EGR1, we examined the circadian expression of clock genes in the livers of *Egr1* KO mice. Overall, the amplitudes of *Bmal1* and *Per1* rhythms were reduced, whereas the amplitude of the *Per2* rhythm was elevated in the livers of *Egr1* KO mice (Fig. 5a). Moreover, the *Rev-erbα* and *Rev-erbβ* expression levels were increased in the livers of *Egr1* KO mice, but the rhythmic expression of the *Rorγ* gene was not altered (Fig. 5a). The *in vivo* data indicated that EGR1 is truly responsible for the expression of clock genes and for the amplitude changes of *Bmal1*, *Per1 and Per2* rhythms. Although the expression levels of the examined genes were impaired in *Egr1*-deficient liver, the phases of these genes appeared to be preserved. A previous report revealed that EGR1 is not involved in the regulation of central clock function^34^. To further exclude the possibility that the perturbation of liver clocks in *Egr1* null mice may be secondary to a compromised central clock, we examined tissue-autonomous clock expression *in vivo*. C57/Bl6J mice were injected with Scrb (Scramble, stable expression of non-targeting siRNA) or *Egr1* siRNA through their tail veins. Indeed, the *Egr1* expression levels were decreased after injection with *Egr1* siRNA (Supplementary Fig. 3a). RNAi knockdown of *Egr1* in the liver decreased the amplitudes of *Bmal1* and *Per1* rhythms while augmenting the *Per2* rhythm (Fig. 5b). *Egr1* knockdown also increased the *Rev-erbα* and *Rev-erbβ* expression levels but did not alter the rhythmic expression of the *Rorγ* gene (Fig. 5b). According to the above-presented results, we also found that the nadirs of *Per1* and *Rev-erbβ* rhythms were advanced approximately 4 hours after knockout or knockdown of *Egr1* (Fig. 5a,b). Thus, we conclude that Egr1 exerts its effects on the circadian clock in a largely tissue-autonomous manner.

### *Per1* gene transcription is activated directly by EGR1 and is responsible for EGR1-regulated clock gene expression

**Figure 6:**
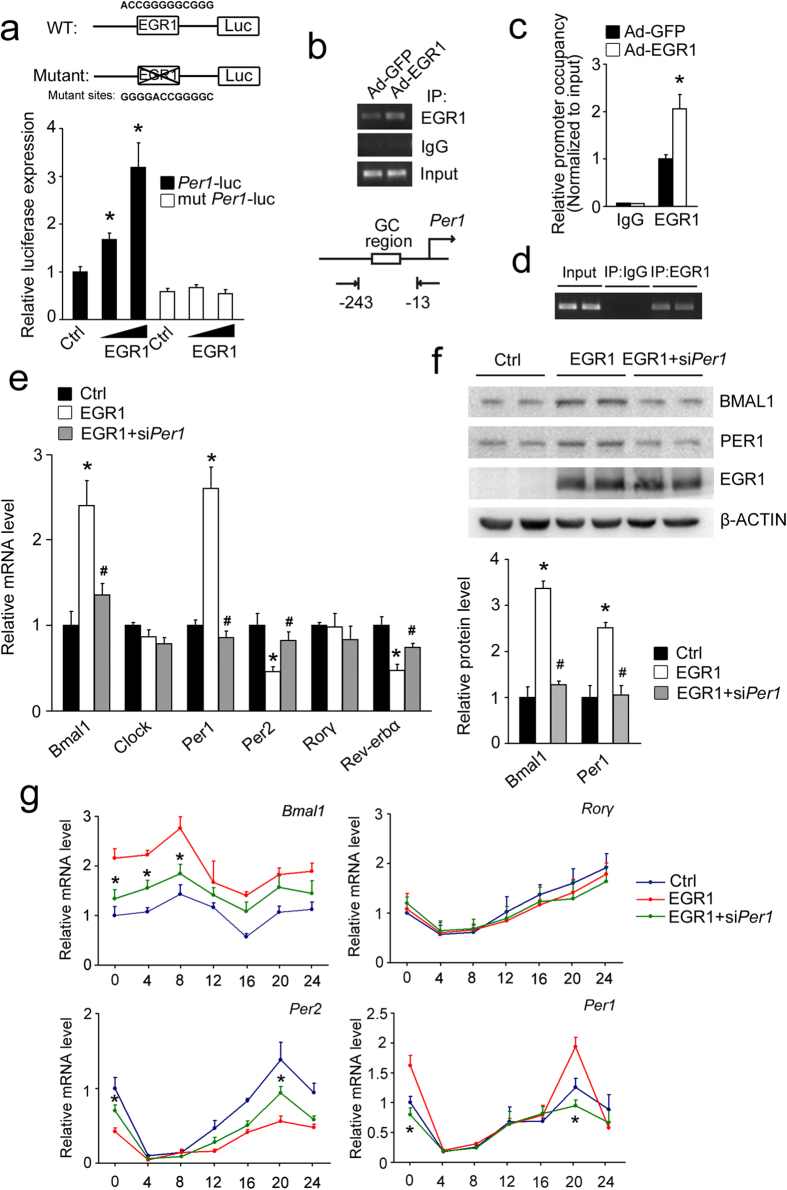
PER1 mediates the regulation of clock genes by EGR1. (**a**) Reporter gene assays in 293T cells using wild-type (*Per1*-luc) or EGR1 binding site mutant (mut *Per1*-luc) *Per1*-luciferase reporters in combination with EGR1 or control plasmid. *P < 0.01, EGR1 versus control. (**b**) ChIP assays with the indicated antibodies using AML-12 cells infected with GFP or EGR1 adenoviruses for 48 h. PCR primers amplified a fragment flanking the GC region on the *Per1* promoter. (**c**) qPCR analysis of ChIP assays with the indicated antibodies. *P < 0.01. (**d**) ChIP assays in mouse livers at ZT13. PCR primers amplify a fragment flanking GC region on the *Per1* promoter. (**e**) qPCR analysis of clock genes in primary hepatocytes infected with the GFP or EGR1 adenoviruses for 48 h after transfection with Scrb or *Per1* siRNA for 6 h. *P < 0.04, EGR1 versus control. ^*#*^*P* < 0.05, EGR1+si*Per1* versus EGR1. (**f**) Protein expression in primary hepatocytes infected with the GFP or EGR1 adenoviruses for 48 h after transfection with Scrb or *Per1* siRNA for 6 h. The gels were run under the same experimental conditions. The full-length blots are presented in Supplementary Fig. 8. *P < 0.02 EGR1 versus control. ^*#*^*P* < 0.02, EGR1+si*Per1* versus EGR1. (**g**) Time course expression of clock genes in mouse AML-12 cells infected with GFP or EGR1 adenoviruses for 48 h after transfection with Scrb or *Per1* siRNA for 6 h following serum shock. *P < 0.05 EGR1+si*Per1* versus EGR1.

We found that a non-canonical EGR1 binding site (GC region, ACCGGGGGCGGG) exists at −90 to −79 of the 5′-flanking sequence of the mouse *Per1* gene. According to the above-presented results, analyzing whether EGR1 activates Per1 transcription directly in mouse liver was reasonable. EGR1 overexpression increased *Per1* promoter reporter activity (Fig. 6a). However, the activating effects of EGR1 were abolished when the EGR1-binding sites on the *Per1* promoter were mutated (Fig. 6a). Chromatin immunoprecipitation (ChIP) assays in mouse AML-12 cells indicated that EGR1 was present near the GC region on the proximal *Per1* promoter and that EGR1 overexpression augmented the binding of EGR1 on the *Per1* promoter (Fig. 6b,c). ChIP assays in mouse liver also revealed that EGR1 was present near the GC region on the proximal *Per1* promoter at ZT13 (Fig. 6d). These results demonstrated that EGR1 activates *Per1* transcription by binding directly to the *Per1* promoter in mouse liver.

We next determined whether PER1 is responsible for the regulation of other clock genes by EGR1 in hepatocytes. We found that the alteration in *Bmal1*, *Per2*, and *Rev-erbα* expression following EGR1 overexpression could be partially restored by *Per1* knockdown (Fig. 6e). *Per1* knockdown also inhibited the upregulation of BMAL1 protein that was induced by EGR1 overexpression (Fig. 6f). Serum shock-induced alterations in the amplitudes of *Bmal1* and *Per2* rhythms following EGR1 overexpression were also partially restored by *Per1* knockdown (Fig. 6g). Collectively, our results confirm that the upregulated expression of Per1 is responsible for the EGR1-regulated amplitude alteration of downstream clock gene rhythms.

### EGR1 is required for the feeding/fasting- or light-induced alteration of clock gene rhythms *in vivo*

**Figure 7:**
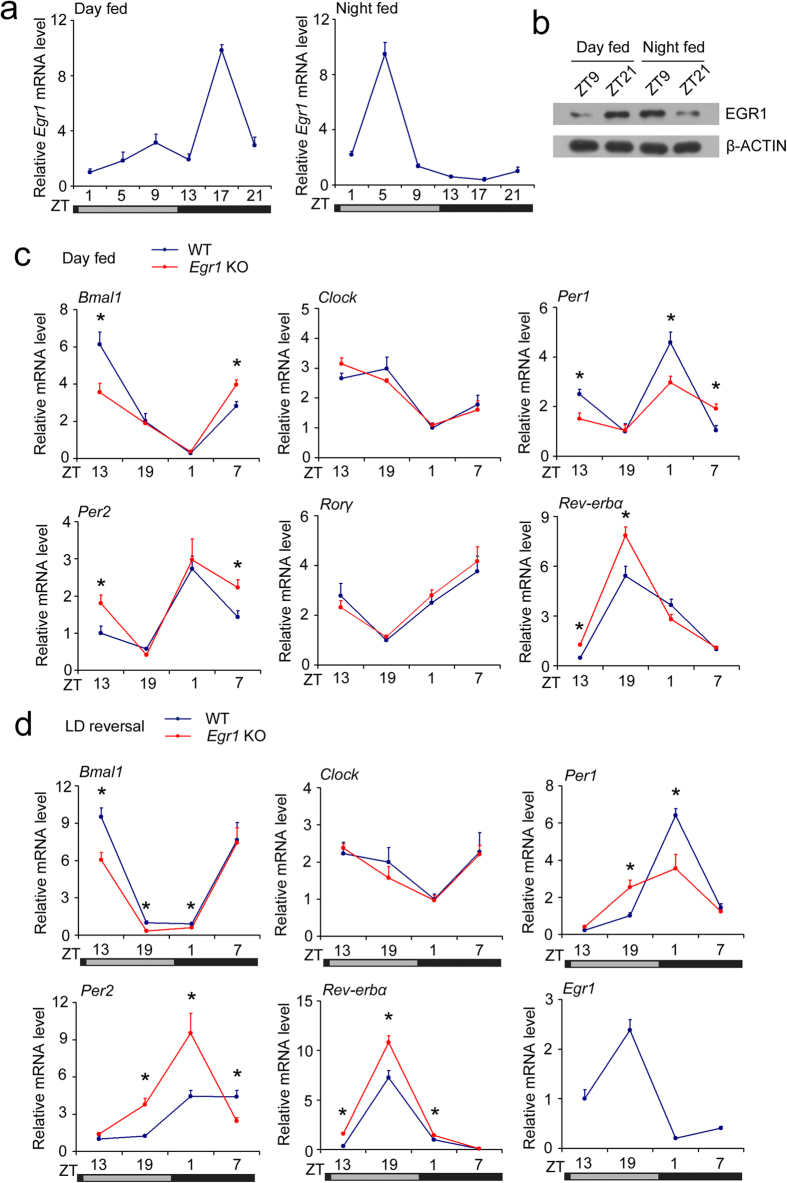
EGR1 mediates the feeding/fasting or light-induced alteration of clock function. (**a**) qPCR analysis of *Egr1* mRNA expression in livers from B6 mice subjected to daytime feeding or nighttime feeding. (**b**) EGR1 protein level at representative timepoints. The gels were run under the same experimental conditions. The full-length blots are presented in Supplementary Fig. 9. (**c**) qRT-PCR analysis of clock gene expression in livers from wild-type and *Egr1* null mice subjected to daytime feeding. (**d**) qRT-PCR analysis of clock gene expression in livers from wild-type and *Egr1* null mice subjected to LD reversal. Data are shown as the mean ± s.d. *P < 0.05, wild-type versus null mice.

The master clock synchronizes peripheral oscillators by imposing light-regulated rest-activity rhythms and feeding-fasting cycles^9^. The feeding-fasting rhythm is a dominant Zeitgeber for peripheral oscillators^35^. We found that the peak of hepatic EGR1 expression was shifted by 12 hours in the day-fed animals compared with the night-fed control animals (Fig. 7a,b). As expected, clock genes such as *Bmal1* and *Per1* were also shifted by 12 hours in the day-fed mice (Supplementary Fig. 4a). Next, we subjected wild-type and *Egr1* null mice to day feeding. Day feeding-induced reset of clock gene rhythms was preserved, but the amplitudes of the rhythms of clock genes, including *Bmal1*, *Per1*, *Per2*, and *Rev-erbα* were reduced significantly in the livers of *Egr1* null mice (Fig. 7c). By contrast, the rhythmic expression of other clock genes (*Clock* and *Rorγ*) was not altered by *Egr1* deletion in the context of day feeding (Fig. 7c). Light:dark (LD) cycle incubation is another model for studying the regulation of peripheral oscillators and master pacemaker. Therefore, we examined the expression patterns of clock genes in the livers of mice after 10 days of LD reversal. We also found that the amplitudes of *Bmal1* and *Per1* rhythms were reduced but that *Per2* and *Rev-erbα* amplitudes were elevated in the livers of *Egr1* null mice after LD reversal (Fig. 7d). Additionally, the rhythmic expression of the *Clock* gene was not altered by *Egr1* deletion (Fig. 7d). These results further confirm that EGR1 modulates the amplitude of hepatic clock gene rhythms.

## Discussion

**Figure 8:**
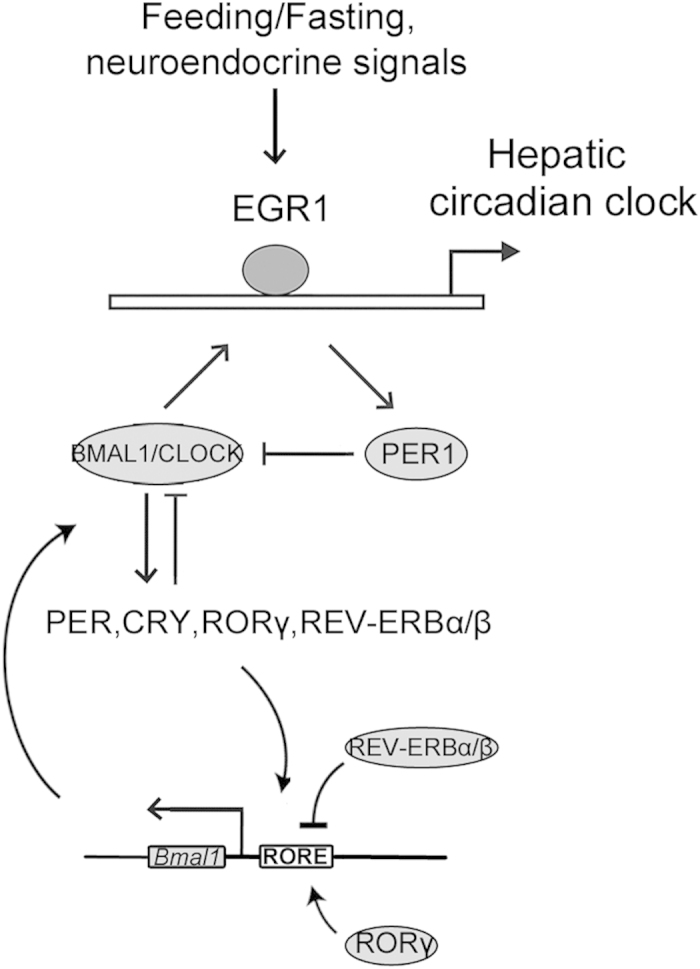
Model for the role of EGR1 in regulating circadian clock function.

As an early growth response factor, EGR1 may mediate the central signals rapidly to maintain proper oscillation of the peripheral clocks. EGR1 is involved in the regulation and development of multiple diseases such as pulmonary inflammation, dilated cardiomyopathy, angiogenesis, insulin resistance and tumorigenesis^36^,^37^,^38^,^39^,^40^,^41^. Our studies revealed that EGR1 is required for the proper amplitudes of the rhythms of several clock gens, including *Bmal1*, *Per1*, *Per2* and *Rev-erbs*. *Egr1* itself is expressed in a circadian manner in the liver and has a phase relationship with components of the core clock gene *Per1*. EGR1 binds to the promoter of the *Per1* gene to activate its transcription. Per1 accumulation may lead to the repression of *Per2* and *Rev-erbs* by inhibiting BMAL1/CLOCK activity. The repression of REV-ERBs may lead to the robust oscillation of *Bmal1*. Furthermore, BMAL1 binds and activates *Egr1* transcription. This feedback loop of EGR1/PER1/BMAL1/EGR1 could potentially help the liver clock keep pace with the master clock (Fig. 8). Although we found the altered expression of *Bmal1*, *Per2* and *Rev-Erbs* by EGR1 is dependent on PER1 activation, whether other factors also mediate the regulation of these clock genes by EGR1 cannot be excluded. Whether EGR1 regulates the expression of other clock genes directly in addition to Per1 also cannot be excluded.

The proper amplitudes of the molecular clock are crucial for the operation of the body’s biological clock, but the significance of the amplitude is not well understood. In *Drosophila*, the circadian amplitude is modulated by temperature and can regulate photoperiodic responses^42^. In *Neurospora*, amplitude variation has been associated with circadian period mutants^43^. In mammals, disruption of circadian amplitude may be related to phase perturbation, instability of circadian rhythms and other homeostatic processes^15^,^16^,^17^,^18^. Previous reports have also shown that high-amplitude rhythms are more difficult to shift than are low-amplitude rhythms^42^. This difficulty in shifting is based on the theory of limit cycle oscillators, where a perturbation of similar strength changes the phase of an oscillator with high amplitude harder than one with lower amplitude because the perturbation represents a larger fraction of the radius of the circle^44^. Vanderleest *et al.*’s findings show that large phase shifts respond in high-amplitude rhythms in slices from short days and small shifts respond in low-amplitude rhythms in slices from long days, which is in contrast to previous reports^45^. These authors have explained that in long day length, with a wide phase distribution, neurons have a more diverse phase shifting response to a light input signal, while in short day length, with a narrow phase distribution, neurons may respond more coherently, resulting in a larger overall shift^45^. According to the previous reports, we hypothesize that the amplitudes of clock gene rhythms might be related to the phase period. A deficiency in EGR1 can affect peripheral clock homeostasis by disrupting the amplitudes of peripheral oscillators. We also found that inhibiting hepatic EGR1 expression altered the phase of *Per1* and *Rev-erbβ* gene rhythms slightly, as shown in Fig. 5a,b. These results suggest that disrupting the oscillation amplitudes of the peripheral clocks by *Egr1* deletion might contribute to the instability of peripheral clock oscillators and increase their sensitivity to phase perturbation. Previous reports also indicate that strong clocks make the oscillatory system more rigid and relax faster in response to a perturbation, while weak clocks make the oscillatory system less rigid and result in the enlargement of the entrainment range^46^,^47^. In old age, the degradation of circadian rhythms is normally accompanied by both loss of amplitude and fragmentation of clock rhythms^17^. Thus, although we could not find published data on *Egr1* levels in elder versus young individuals, we speculate that disruption of *Egr1* oscillation may mediate the age-induced deterioration of peripheral clock rhythms.

Perturbed clock function has been implicated in sleep disorders and is associated with an increased risk of metabolic diseases. Epidemiological studies have revealed that shift workers are more predisposed to elevated triglycerides, HDL cholesterol levels and obesity than day workers^48^. Remarkably, the disruption of clock function in rodents leads to obesity and impaired glucose, lipid and cholesterol homeostasis. For example, *Clock* mutant mice present obesity, dyslipidemia and hepatic steatosis^49^, and *Bmal1* null mice display reduced glucose tolerance and elevated circulating FFA and cholesterol levels^50^. Certain nuclear factors, including PPARs, PGC1α, GR and BAF60A, can link the metabolic pathways and the circadian clock at the transcriptional level^51^,^52^,^53^. Thus, the relationship between the circadian clock and these metabolic pathways would represent a novel target for studies of the pathogenesis of metabolic diseases.

EGR1 has been implicated in the regulation of key metabolic processes, including adipocyte insulin resistance, energy storage, obesity development and insulin biosynthesis^39^,^40^,^54^,^55^. EGR1 has been reported to regulate hepatic gluconeogenesis and cholesterol biosynthesis in the liver^24^,^25^. Adipocyte EGR1 was reported to regulate insulin resistance, energy expenditure and obesity^54^. Our results also show that *Egr1* is expressed in a circadian manner in WAT. Further studied are required to elucidate whether EGR1 is involved in the integration of the circadian clock and whole-body energy metabolism. Time-restricted feeding was reported to prevent metabolic diseases in mice that were fed a high-fat diet and represents a preventative and therapeutic intervention against diverse nutritional challenges^56^,^57^. These reports also suggest that EGR1 expression may orchestrate these metabolic processes during time-restricted feeding.

In conclusion, we have identified that EGR1, an early growth response factor, is a critical transcription factor that regulates the hepatic clock circuit. Investigating the role of EGR1 in the integration of the circadian clock and whole-body energy metabolism could be interesting in future studies.

## Methods

### Materials

Anti-EGR1 antibody was purchased from Santa Cruz Biotech, anti-BMAL1 and anti-CLOCK antibodies were obtained from Abcam, anti-PER1 antibody was obtained from Pierce, anti-GAPDH antibody was obtained from KangChen Biotech (Shanghai, China), and anti-β-ACTIN antibody was obtained from Boster Biotech (Wuhan, China). Invivofectamine 2.0 reagent was obtained from Invitrogen. The donor equine serum was purchased from Hyclone.

### mRNA and protein expression analysis

Total RNA was isolated using TRIzol reagent (TaKaRa), reverse transcribed, and analyzed by quantitative PCR (qPCR) using SYBR Green and an ABI 7300 system (Applied Biosystems, Carlsbad, CA). A complete list of PCR primers is shown in Supplementary Table 1. For protein analysis, we lysed cells or homogenized tissues directly in RIPA buffer, and then centrifuged them at 4 °C for 10 min at 13,200 × g. The resulting supernatant fraction was separated by SDS-PAGE and immunoblotted with the indicated antibodies. Immunoreactivity was detected using Kodak X-OMAT film (Kodak) or a MiniChemi Imager (SageCreation, Beijing, China). The intensities of the bands were quantified using NIH ImageJ software. All the proteins were normalized to the internal control β-ACTIN or GAPDH.

### Animal experiments

All animal experiments were performed after the approval from the the Laboratory Animal Care Committee at Nanjing University. Animal welfare and experimental procedures were carried out in accordance with the Guide for the Care and Use of Laboratory Animals (Ministry of Science and Technology of China, 2006) and related ethical regulations of Nanjing University. *Egr1* KO mice were produced as described previously^58^. All mice used in this study were males at 10–12 weeks of age. To analyze EGR1 expression in various tissues, C57/Bl6J (B6) mice were housed in a 12-h light/12-h dark cycle (light/dark, 12:12) in a temperature- and humidity-controlled environment and were fed ad libitum. Zeitgeber time zero (ZT0) referred to lights on time. Tissues from five mice were dissected every 4 h for 24 h and subsequently processed for qRT-PCR and immunoblot analysis. To analyze gene expression in wild-type and *Egr1* null mice, five mice of each genotype were euthanized every six hours. Tissues were immediately frozen and subsequently processed for qPCR. To analyze gene expression in wild-type and *Bmal1* null mice, the mice were kept under light:dark (LD) 12:12 h and subsequently subjected to constant darkness for 36 h. To analyze gene expression after alterations of light cues, the mice were transferred to a completely reversed LD cycle for ten days. For restricted feeding, the mice were fed exclusively at night or day for ten days, and then the livers from five mice were dissected every 4 h for 24 h and subsequently processed for qRT-PCR.

### siRNA injection *in vivo*

B6 mice were injected intravenously with 200 μL of *Egr1* siRNA or Scrb siRNA (70 mg) and Invivofectamine 2.0 reagent (Invitrogen). To ensure that the siRNA was concentrated in the liver, the siRNA was labeled by cholesterol^59^. The use of Invivofectamine 2.0 as the transfection reagent *in vivo* can further increase the concentration of siRNA in the liver without affecting other tissues. The mice were injected twice at 4-day intervals (8 mg/kg) and were then sacrificed 4 days after the last injection at the indicated timepoints. siRNA sequences are shown in Supplementary Table 1.

### siRNA and plasmid transfection

For siRNA transfection, primary hepatocytes or mouse AML-12 hepatocytes were transfected siRNA based on a Scrb sequence or with siRNA specific for *Per1*, using Lipofectamine 2000 (Invitrogen) according to the manufacturer’s protocols. siRNA sequences are shown in Supplementary Table 1. For plasmid transfection, mouse AML-12 hepatocytes were transfected with BMAL1 and CLOCK plasmids or control PCDNA3 plasmid, cells were processed for qRT-PCR or immunoblot analysis after 48 hours of transfection.

### Serum shock

The mouse liver cell line AML-12 was transduced with corresponding adenoviruses or siRNA and was then established and maintained in DMEM/F12 supplemented with 10% FBS. For serum shock, media of confluent cultures were replaced with DMEM/F12 plus 50% horse serum (t = 0). After 2 h, the cells were washed once with PBS and incubated with serum-free medium. Total RNA was extracted at the indicated time points and processed for qRT-PCR analysis using 36B4 as a normalization control.

### Reporter gene assays

Reporter gene assays were performed in 293T cells. In a typical experiment, 100 ng of *Per1* reporter plasmid or mutant plasmid was mixed with 400 or 800 ng of the expression construct for EGR1. Equal amounts of DNA were used for all transfection combinations by adding the appropriate vector DNA. To control for transfection efficiency, cells were co-transfected with the PRL plasmid (10 ng). Relative luciferase activities were determined 48 h following transfection using the Dual-Luciferase assay system (Promega) according to the manufacturer’s protocol. All transfection experiments were performed in triplicate.

### ChIP assay

Chromatin immunoprecipitation was performed essentially as described by Upstate Biotechnology. Briefly, mouse AML-12 cells were injected with GFP or EGR1 adenoviruses for 48 h. For *in vivo* ChIP, mouse liver samples were collected at the corresponding timepoints. Chromatin lysates were prepared, pre-cleared with Protein-A/G agarose beads, and immunoprecipitated with antibodies against the corresponding protein or control mouse IgG (Santa Cruz Biotech) in the presence of BSA and salmon sperm DNA. Beads were extensively washed before reverse cross-linking. DNA was purified using a PCR purification kit (Qiagen) and subsequently analyzed by PCR or qPCR.

### Statistical analysis

All the experiments were performed in triplicate. The data are presented as the mean ± S.D. We performed statistical comparisons with unpaired two-tailed Student’s *t*-test. *P* < 0.05 was considered statistically significant.

## Additional Information

**How to cite this article**: Tao, W. *et al.* EGR1 regulates hepatic clock gene amplitude by activating *Per1* transcription. *Sci. Rep.* **5**, 15212; doi: 10.1038/srep15212 (2015).

## Supplementary Material

Supplementary Information

## References

[b1] King D. P. & Takahashi J. S. Molecular genetics of circadian rhythms in mammals. Annu Rev Neurosci 23, 713–742 (2000). 10845079 10.1146/annurev.neuro.23.1.713

[b2] Lowrey P. L. & Takahashi J. S. Genetics of the mammalian circadian system: Photic entrainment, circadian pacemaker mechanisms, and posttranslational regulation. Annu Rev Genet 34, 533–562 (2000). 11092838 10.1146/annurev.genet.34.1.533

[b3] Reppert S. M. & Weaver D. R. Coordination of circadian timing in mammals. Nature 418, 935–941 (2002). 12198538 10.1038/nature00965

[b4] Rutter J., Reick M. & McKnight S. L. Metabolism and the control of circadian rhythms. Annu Rev Biochem 71, 307–331 (2002). 12045099 10.1146/annurev.biochem.71.090501.142857

[b5] Tu B. P. & McKnight S. L. Metabolic cycles as an underlying basis of biological oscillations. Nat Rev Mol Cell Biol 7, 696–701 (2006). 16823381 10.1038/nrm1980

[b6] Wijnen H. & Young M. W. Interplay of circadian clocks and metabolic rhythms. Annu Rev Genet 40, 409–448 (2006). 17094740 10.1146/annurev.genet.40.110405.090603

[b7] Maury E., Ramsey K. M. & Bass J. Circadian rhythms and metabolic syndrome: from experimental genetics to human disease. Circ Res 106, 447–462 (2010). 20167942 10.1161/CIRCRESAHA.109.208355 PMC2837358

[b8] Korencic A. *et al.* Timing of circadian genes in mammalian tissues. Sci Rep 4, 5782 (2014). 25048020 10.1038/srep05782 PMC5376044

[b9] Schibler U., Ripperger J. & Brown S. A. Peripheral circadian oscillators in mammals: time and food. J Biol Rhythms 18, 250–260 (2003). 12828282 10.1177/0748730403018003007

[b10] Schibler U. & Sassone-Corsi P. A web of circadian pacemakers. Cell 111, 919–922 (2002). 12507418 10.1016/s0092-8674(02)01225-4

[b11] Zylka M. J. *et al.* Molecular analysis of mammalian timeless. Neuron 21, 1115–1122 (1998). 9856466 10.1016/s0896-6273(00)80628-5

[b12] Kume K. *et al.* mCRY1 and mCRY2 are essential components of the negative limb of the circadian clock feedback loop. Cell 98, 193–205 (1999). 10428031 10.1016/s0092-8674(00)81014-4

[b13] Preitner N. *et al.* The orphan nuclear receptor REV-ERBalpha controls circadian transcription within the positive limb of the mammalian circadian oscillator. Cell 110, 251–260 (2002). 12150932 10.1016/s0092-8674(02)00825-5

[b14] Sato T. K. *et al.* A functional genomics strategy reveals Rora as a component of the mammalian circadian clock. Neuron 43, 527–537 (2004). 15312651 10.1016/j.neuron.2004.07.018

[b15] Johnson C. H. Forty years of PRCs—what have we learned? Chronobiol Int 16, 711–743 (1999). 10584173 10.3109/07420529909016940

[b16] Vitaterna M. H. *et al.* The mouse Clock mutation reduces circadian pacemaker amplitude and enhances efficacy of resetting stimuli and phase-response curve amplitude. Proc Natl Acad Sci USA 103, 9327–9332 (2006). 16754844 10.1073/pnas.0603601103 PMC1474012

[b17] Hofman M. A. & Swaab D. F. Living by the clock: the circadian pacemaker in older people. Ageing Res Rev 5, 33–51 (2006). 16126012 10.1016/j.arr.2005.07.001

[b18] Monk T. H. Aging human circadian rhythms: conventional wisdom may not always be right. J Biol Rhythms 20, 366–374 (2005). 16077155 10.1177/0748730405277378

[b19] Hamilton T. B., Borel F. & Romaniuk P. J. Comparison of the DNA binding characteristics of the related zinc finger proteins WT1 and EGR1. Biochemistry 37, 2051–2058 (1998). 9485332 10.1021/bi9717993

[b20] Silverman E. S. & Collins T. Pathways of Egr-1-mediated gene transcription in vascular biology. Am J Pathol 154, 665–670 (1999). 10079243 10.1016/S0002-9440(10)65312-6 PMC1866415

[b21] Mostecki J., Showalter B. M. & Rothman P. B. Early growth response-1 regulates lipopolysaccharide-induced suppressor of cytokine signaling-1 transcription. J Biol Chem 280, 2596–2605 (2005). 15545275 10.1074/jbc.M408938200

[b22] Yao J., Mackman N., Edgington T. S. & Fan S. T. Lipopolysaccharide induction of the tumor necrosis factor-alpha promoter in human monocytic cells. Regulation by Egr-1, c-Jun, and NF-kappaB transcription factors. J Biol Chem 272, 17795–17801 (1997). 9211933 10.1074/jbc.272.28.17795

[b23] Virolle T. *et al.* The Egr-1 transcription factor directly activates PTEN during irradiation-induced signalling. Nat Cell Biol 3, 1124–1128 (2001). 11781575 10.1038/ncb1201-1124

[b24] Shen N. *et al.* The Constitutive Activation of Egr-1/C/EBPa Mediates the Development of Type 2 Diabetes Mellitus by Enhancing Hepatic Gluconeogenesis. Am J Pathol 185, 513–523 (2015). 25438063 10.1016/j.ajpath.2014.10.016

[b25] Gokey N. G., Lopez-Anido C., Gillian-Daniel A. L. & Svaren J. Early growth response 1 (Egr1) regulates cholesterol biosynthetic gene expression. J Biol Chem 286, 29501–29510 (2011). 21712389 10.1074/jbc.M111.263509 PMC3190990

[b26] Kim S. H. *et al.* Egr1 regulates lithium-induced transcription of the Period 2 (PER2) gene. Biochim Biophys Acta 1832, 1969–1979 (2013). 23816566 10.1016/j.bbadis.2013.06.010

[b27] Fustin J. M. *et al.* Egr1 involvement in evening gene regulation by melatonin. FASEB J 23, 764–773 (2009). 19019852 10.1096/fj.08-121467

[b28] Resuehr H. E., Resuehr D. & Olcese J. Induction of mPer1 expression by GnRH in pituitary gonadotrope cells involves EGR-1. Mol Cell Endocrinol 311, 120–125 (2009). 19616057 10.1016/j.mce.2009.07.005

[b29] Panda S. *et al.* Coordinated transcription of key pathways in the mouse by the circadian clock. Cell 109, 307–320 (2002). 12015981 10.1016/s0092-8674(02)00722-5

[b30] Hughes M. E. *et al.* Harmonics of circadian gene transcription in mammals. PLoS Genet 5, e1000442 (2009). 19343201 10.1371/journal.pgen.1000442 PMC2654964

[b31] Yan J., Wang H., Liu Y. & Shao C. Analysis of gene regulatory networks in the mammalian circadian rhythm. PLoS Comput Biol 4, e1000193 (2008). 18846204 10.1371/journal.pcbi.1000193 PMC2543109

[b32] Bozek K. *et al.* Regulation of clock-controlled genes in mammals. PLoS One 4, e4882 (2009). 19287494 10.1371/journal.pone.0004882 PMC2654074

[b33] Balsalobre A., Damiola F. & Schibler U. A serum shock induces circadian gene expression in mammalian tissue culture cells. Cell 93, 929–937 (1998). 9635423 10.1016/s0092-8674(00)81199-x

[b34] Kilduff T. S. *et al.* Characterization of the circadian system of NGFI-A and NGFI-A/NGFI-B deficient mice. J Biol Rhythms 13, 347–357 (1998). 9711509 10.1177/074873098129000174

[b35] Damiola F. *et al.* Restricted feeding uncouples circadian oscillators in peripheral tissues from the central pacemaker in the suprachiasmatic nucleus. Genes Dev 14, 2950–2961 (2000). 11114885 10.1101/gad.183500 PMC317100

[b36] Shen N. *et al.* Cigarette smoke-induced pulmonary inflammatory responses are mediated by EGR-1/GGPPS/MAPK signaling. Am J Pathol 178, 110–118 (2011). 21224049 10.1016/j.ajpath.2010.11.016 PMC3069843

[b37] Lucas T. *et al.* Overexpression of Egr-1 is associated with dilated cardiomyopathy and induces cardiac cell apoptosis. The FASEB Journal 21, A13 (2007).

[b38] Khachigian L. M. Early growth response-1: blocking angiogenesis by shooting the messenger. Cell Cycle 3, 10–11 (2004). 14657654

[b39] Shen N. *et al.* An early response transcription factor, Egr-1, enhances insulin resistance in type 2 diabetes with chronic hyperinsulinism. Journal of Biological Chemistry 286, 14508–14515 (2011). 21321112 10.1074/jbc.M110.190165 PMC3077649

[b40] Yu X. *et al.* Egr‐1 decreases adipocyte insulin sensitivity by tilting PI3K/Akt and MAPK signal balance in mice. The EMBO journal 30, 3754–3765 (2011). 21829168 10.1038/emboj.2011.277 PMC3173797

[b41] Abdulkadir S. A. *et al.* Impaired prostate tumorigenesis in Egr1-deficient mice. Nat Med 7, 101–107 (2001). 11135623 10.1038/83231

[b42] Pittendrigh C. S., Kyner W. T. & Takamura T. The amplitude of circadian oscillations: temperature dependence, latitudinal clines, and the photoperiodic time measurement. J Biol Rhythms 6, 299–313 (1991). 1773097 10.1177/074873049100600402

[b43] Lakin-Thomas P. L., Brody S. & Cote G. G. Amplitude model for the effects of mutations and temperature on period and phase resetting of the Neurospora circadian oscillator. J Biol Rhythms 6, 281–297 (1991). 1837742 10.1177/074873049100600401

[b44] Aschoff J. & Pohl H. Phase relations between a circadian rhythm and its zeitgeber within the range of entrainment. Naturwissenschaften 65, 80–84 (1978). 345129 10.1007/BF00440545

[b45] vanderLeest H. T., Rohling J. H., Michel S. & Meijer J. H. Phase shifting capacity of the circadian pacemaker determined by the SCN neuronal network organization. PLoS One 4, e4976 (2009). 19305510 10.1371/journal.pone.0004976 PMC2655235

[b46] Abraham U. *et al.* Coupling governs entrainment range of circadian clocks. Mol Syst Biol 6, 438 (2010). 21119632 10.1038/msb.2010.92 PMC3010105

[b47] Granada A. E., Bordyugov G., Kramer A. & Herzel H. Human chronotypes from a theoretical perspective. PLoS One 8, e59464 (2013). 23544070 10.1371/journal.pone.0059464 PMC3609763

[b48] Karlsson B., Knutsson A. & Lindahl B. Is there an association between shift work and having a metabolic syndrome? Results from a population based study of 27,485 people. Occup Environ Med 58, 747–752 (2001). 11600731 10.1136/oem.58.11.747 PMC1740071

[b49] Turek F. W. *et al.* Obesity and metabolic syndrome in circadian Clock mutant mice. Science 308, 1043–1045 (2005). 15845877 10.1126/science.1108750 PMC3764501

[b50] Shimba S. *et al.* Deficient of a clock gene, brain and muscle Arnt-like protein-1 (BMAL1), induces dyslipidemia and ectopic fat formation. PLoS One 6, e25231 (2011). 21966465 10.1371/journal.pone.0025231 PMC3178629

[b51] Liu C., Li S., Liu T., Borjigin J. & Lin J. D. Transcriptional coactivator PGC-1alpha integrates the mammalian clock and energy metabolism. Nature 447, 477–481 (2007). 17476214 10.1038/nature05767

[b52] Tao W. *et al.* SWItch/sucrose nonfermentable (SWI/SNF) complex subunit BAF60a integrates hepatic circadian clock and energy metabolism. Hepatology 54, 1410–1420 (2011). 21725993 10.1002/hep.24514

[b53] Lin J. D., Liu C. & Li S. Integration of energy metabolism and the mammalian clock. Cell Cycle 7, 453–457 (2008). 18235232 10.4161/cc.7.4.5442

[b54] Zhang J. *et al.* Dietary obesity-induced Egr-1 in adipocytes facilitates energy storage via suppression of FOXC2. Sci Rep 3, 1476 (2013). 23502673 10.1038/srep01476 PMC3600596

[b55] Muller I., Rossler O. G., Wittig C., Menger M. D. & Thiel G. Critical role of Egr transcription factors in regulating insulin biosynthesis, blood glucose homeostasis, and islet size. Endocrinology 153, 3040–3053 (2012). 22597533 10.1210/en.2012-1064

[b56] Chaix A., Zarrinpar A., Miu P. & Panda S. Time-restricted feeding is a preventative and therapeutic intervention against diverse nutritional challenges. Cell Metab 20, 991–1005 (2014). 25470547 10.1016/j.cmet.2014.11.001 PMC4255155

[b57] Hatori M. *et al.* Time-restricted feeding without reducing caloric intake prevents metabolic diseases in mice fed a high-fat diet. Cell Metab 15, 848–860 (2012). 22608008 10.1016/j.cmet.2012.04.019 PMC3491655

[b58] Wu X. *et al.* Mechano-sensitive transcriptional factor Egr-1 regulates insulin-like growth factor-1 receptor expression and contributes to neointima formation in vein grafts. Arterioscler Thromb Vasc Biol 30, 471–476 (2010). 19965784 10.1161/ATVBAHA.109.184259

[b59] Wolfrum C. *et al.* Mechanisms and optimization of *in vivo* delivery of lipophilic siRNAs. Nat Biotechnol 25, 1149–1157 (2007). 17873866 10.1038/nbt1339

