# Supplementary material for: EGR1 regulates hepatic clock gene amplitude by activating Per1 transcription
Source: Sci Rep. 2015 Oct 16;5:15212. doi: 10.1038/srep15212 (PMC4607941; doi:10.1038/srep15212)
Supplement: Supplementary Information [file srep15212-s1.pdf]

Supplementary section to:

EGR1 regulates hepatic clock gene amplitude by activating *Per1* transcription

Weiwei Tao[1], Jing Wu[1], Qian Zhang[1], Shan-Shan Lai[1], Shan Jiang[1], Chen Jiang[1], Ying Xu[1], Bin Xue[1], Jie Du[2]*, Chao-Jun Li[1]*

[1]MOE Key Laboratory of Model Animals for Disease Study, Model Animal Research Center (MARC) and the School of Medicine, Nanjing University, Nanjing 210093, China,

[2]Beijing Anzhen Hospital, Capital Medical University, Beijing, 100029, China.

*Correspondence and requests for materials should be addressed to J. D. (jdu@bcm.edu) or C.J.L. (licj@nju.edu.cn).

Supplementary Figures

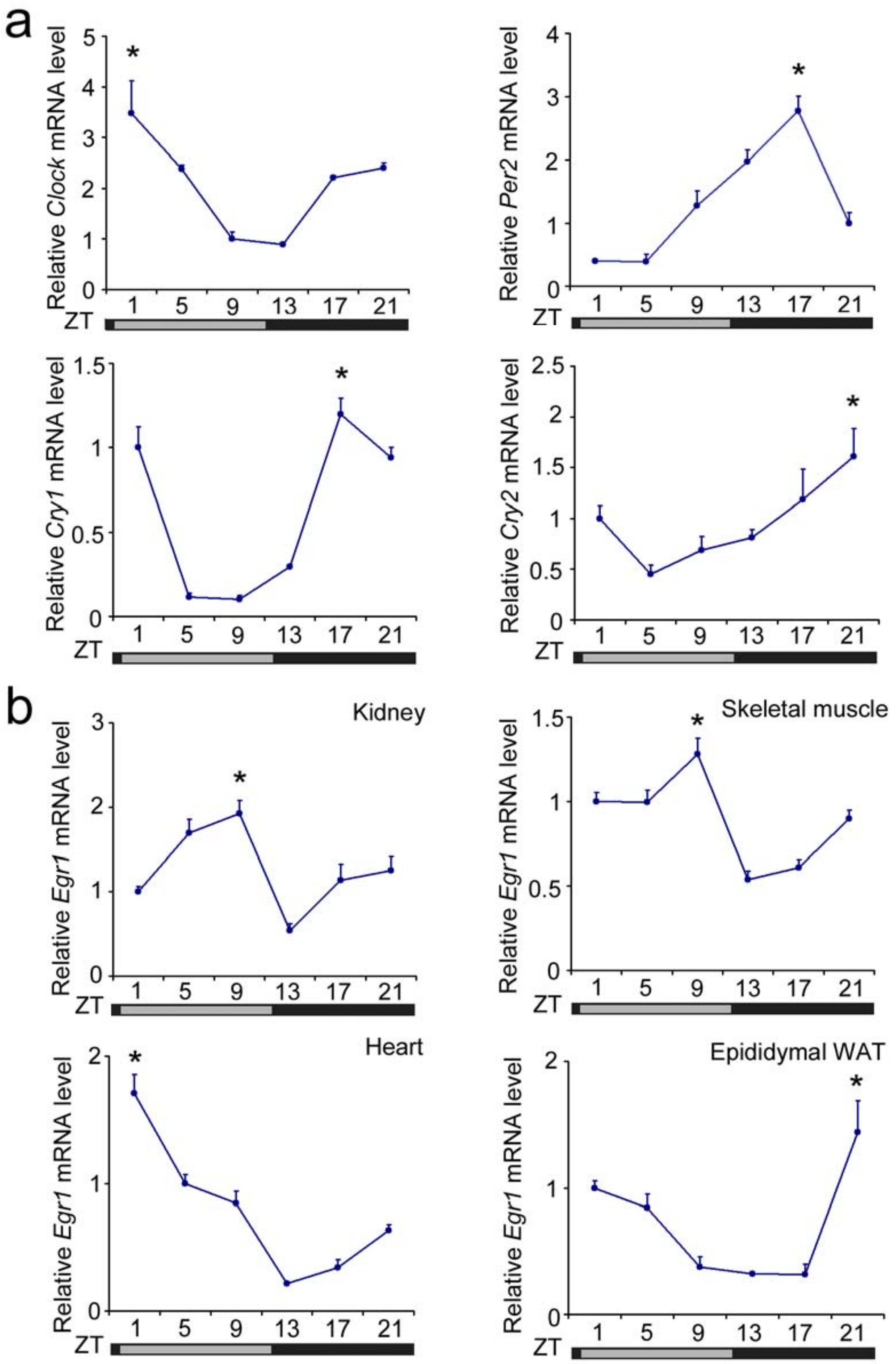

Supplementary Figure 1. Diurnal expression of clock genes and *Egr1* in mouse tissues.

(a) qRT-PCR analysis of clock gene expression in the livers from B6 mice entrained to an LD 12:12 cycle. (b) qRT-PCR analysis of *Egr1* mRNA expression in the kidney, skeletal muscle, heart, and white adipose tissue (WAT) from B6 mice entrained to an LD 12:12 cycle. Data are shown as the mean ± s.d. *P<0.04, peak versus nadir.

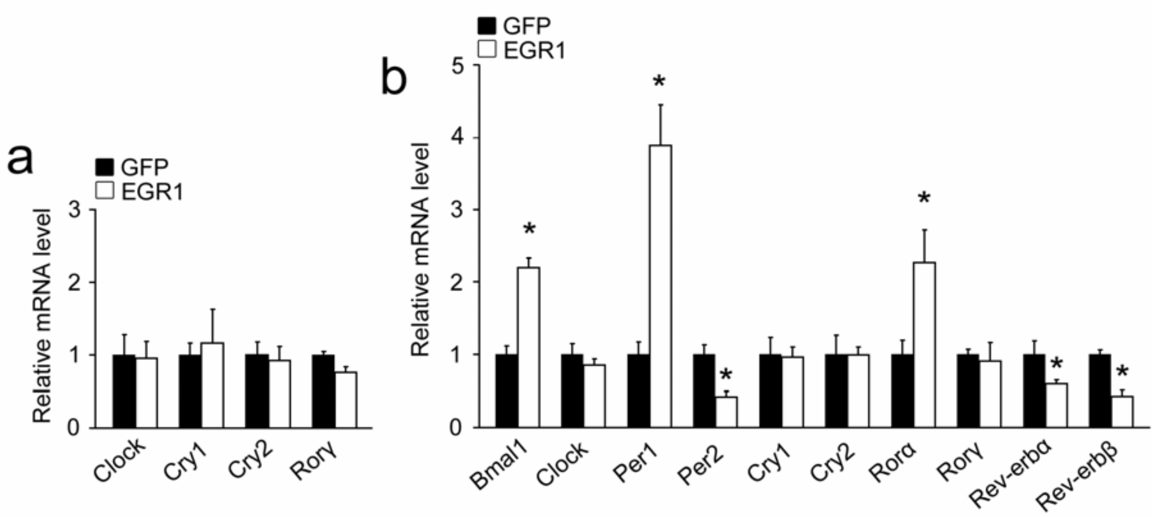

Supplementary Figure 2. Clock gene expression after EGR1 overexpression in hepatocytes.

(a) qPCR analysis of clock genes in primary hepatocytes infected with GFP or EGR1 adenoviruses for 48 h. (b) qPCR analysis of clock genes in mouse AML-12 cells infected with GFP or EGR1 adenoviruses for 48 h. Data are shown as the mean ± s.d. *P<0.03.

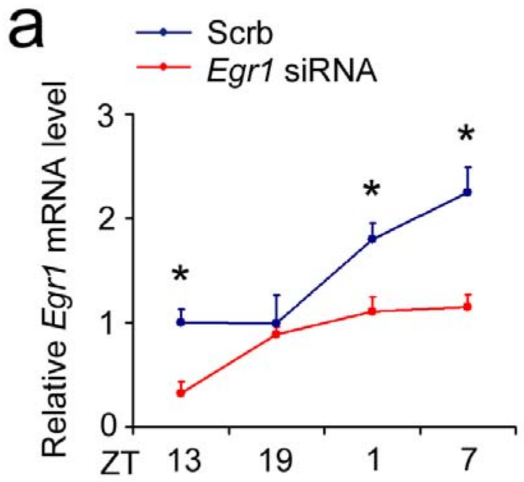

Supplementary Figure 3. Knockdown efficiency of *Egr1* in the livers from B6 mice injected with Scrb or *Egr1* siRNA.

(a) qRT-PCR analysis of *Egr1* mRNA expression in the livers from B6 mice injected with Scrb or *Egr1* siRNA through tail vein injections. **P*<0.05, *Egr1* siRNA versus Scrb.

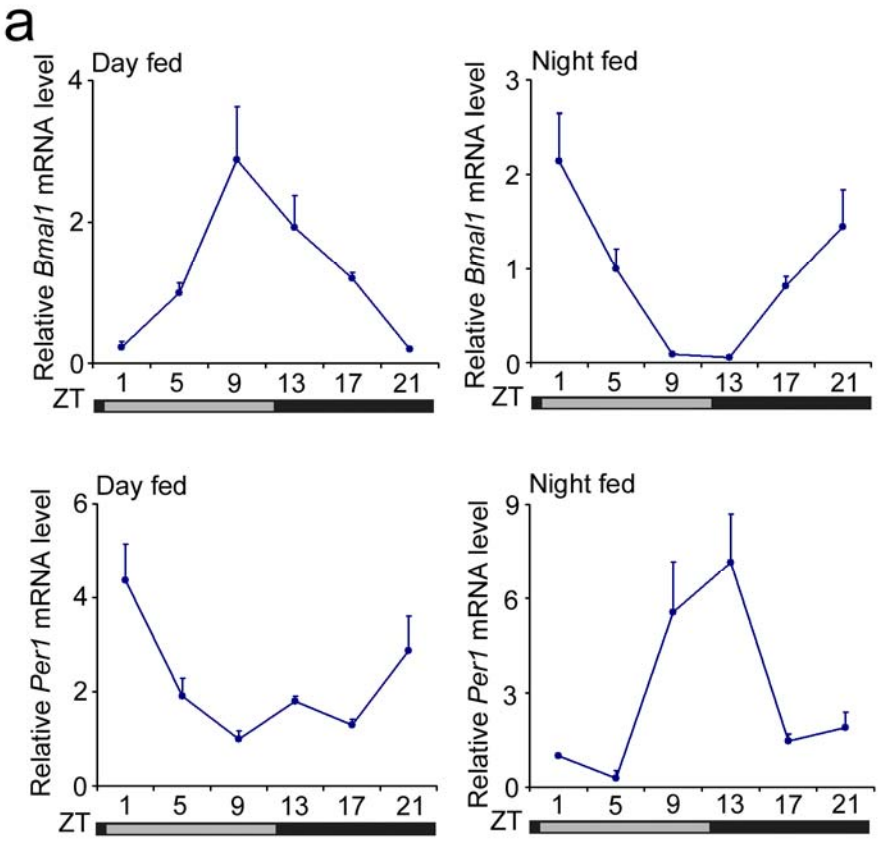

Supplementary Figure 4. Restricted feeding induces phase reset of *Bmal1* and *Per1* genes

(a) qPCR analysis of *Bmal1* and *Per1* mRNA expression in the livers from B6 mice subjected to daytime or nighttime feeding. Data are shown as the mean ± s.d.

Figure 1b

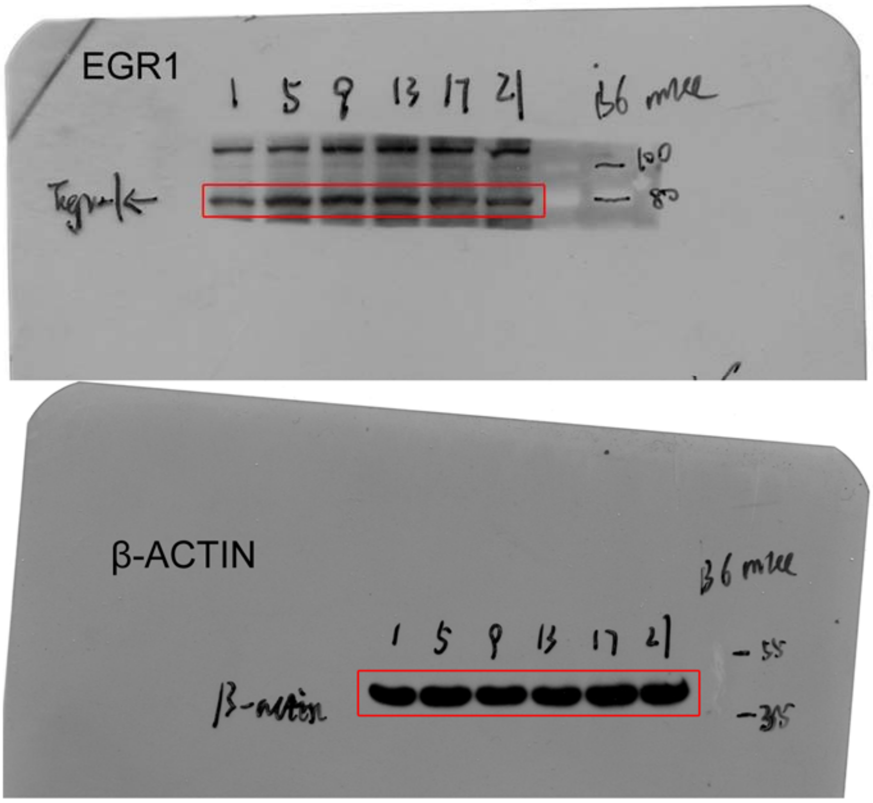

Supplementary Figure 5. Full-length images of the immunoblots in Figure 1b.

Red line boxes indicate the cropped images used in Figure 1b. β-ACTIN was used as an internal control.

Figure 2d

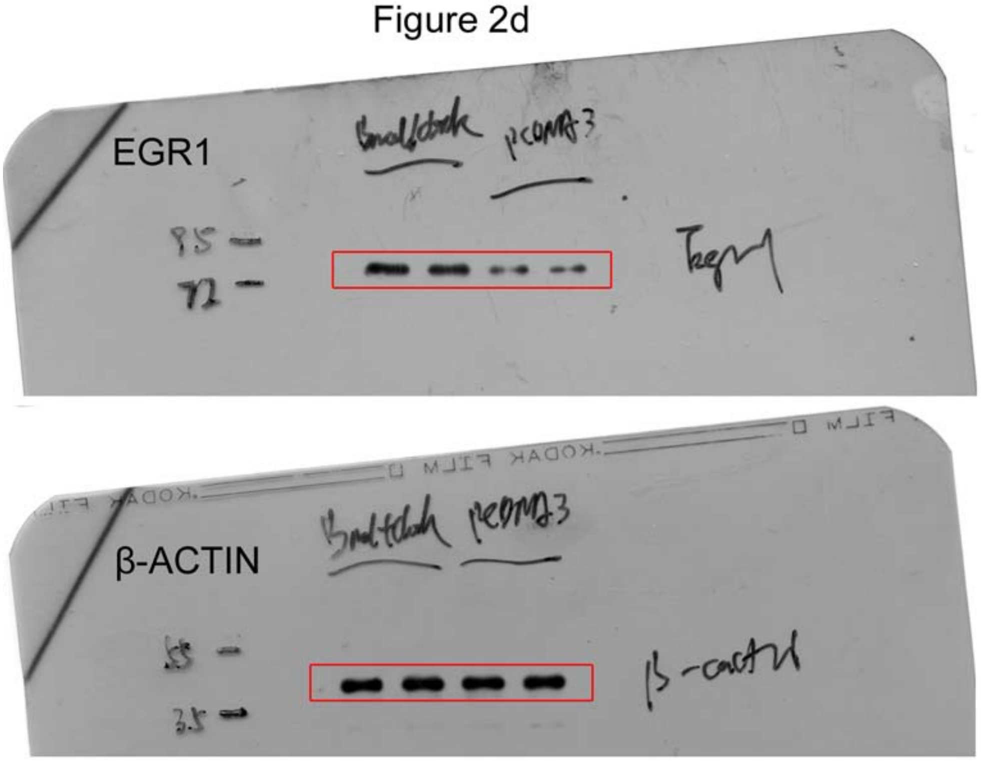

Supplementary Figure 6. Full-length images of the immunoblots in Figure 2d.

Red line boxes indicate the cropped images used in Figure 2d. β-ACTIN was used as an internal control.

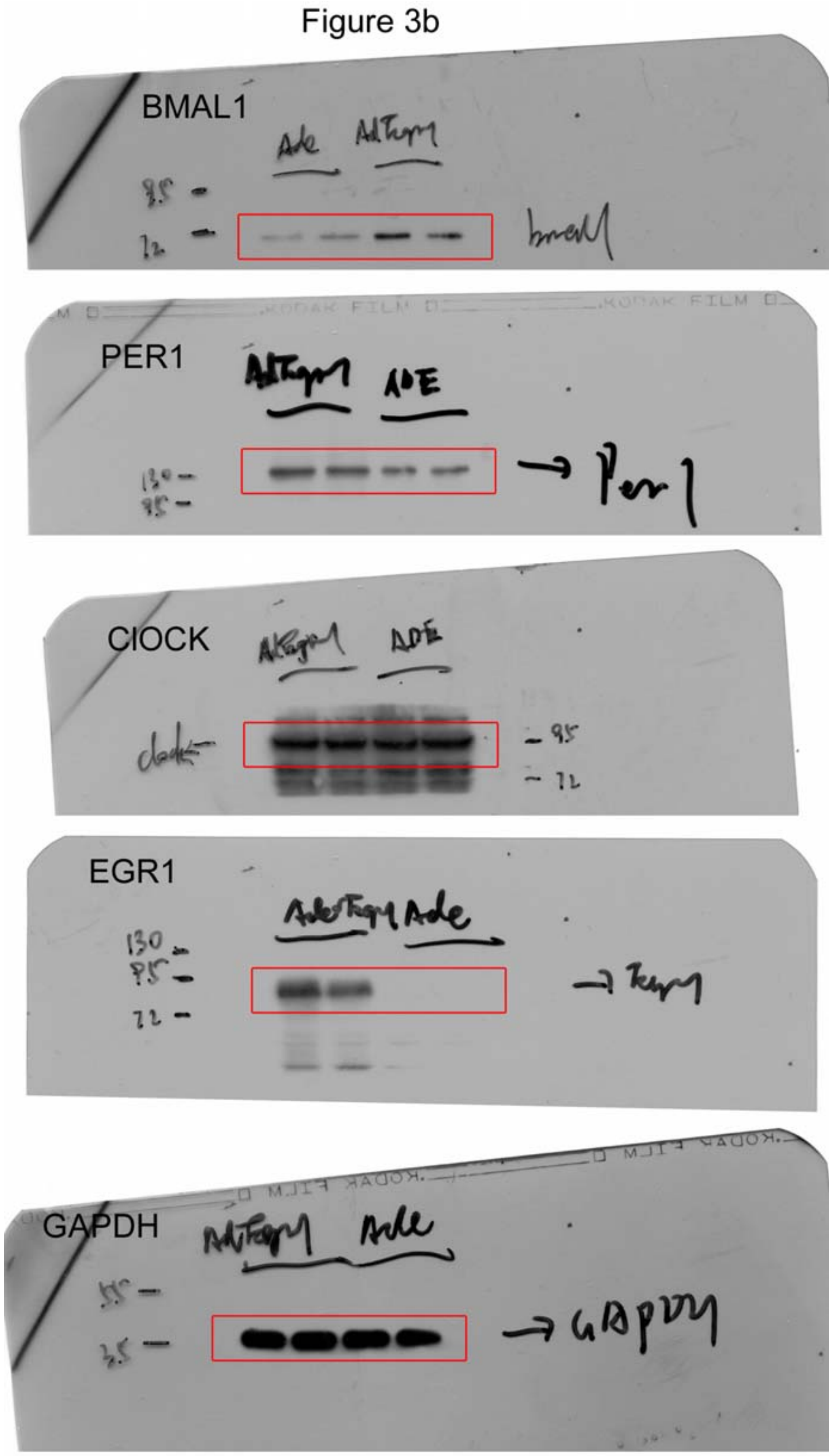

Supplementary Figure 7. Full-length images of the immunoblots in Figure 3b.

Red line boxes indicate the cropped images used in Figure 3b. GAPDH was used as an internal control.

Figure 6f

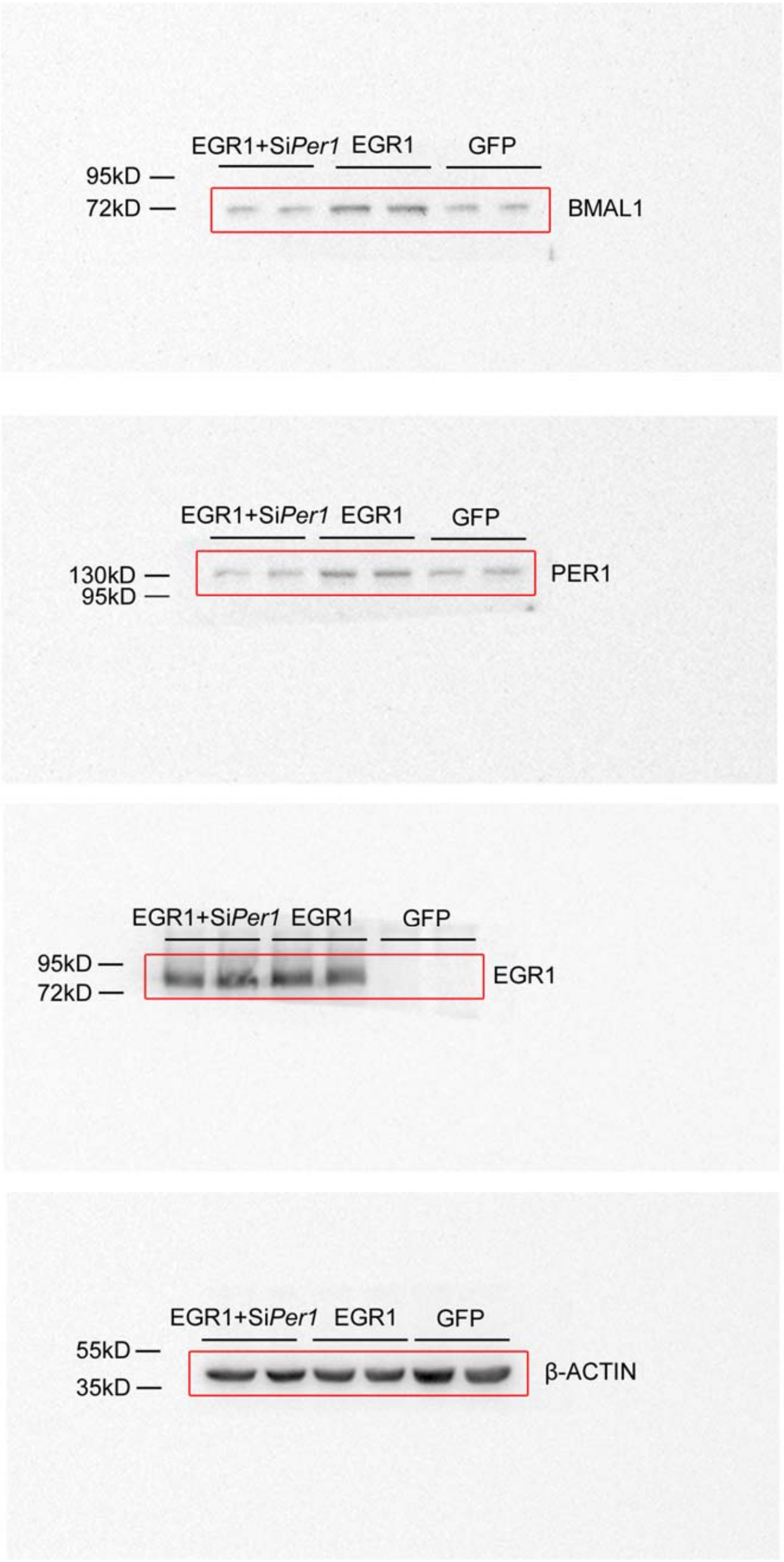

Supplementary Figure 8. Full-length images of the immunoblots in Figure 6f.

Red line boxes indicate the cropped images used in Figure 6f. β-ACTIN was used as an internal control.

Figure 7b

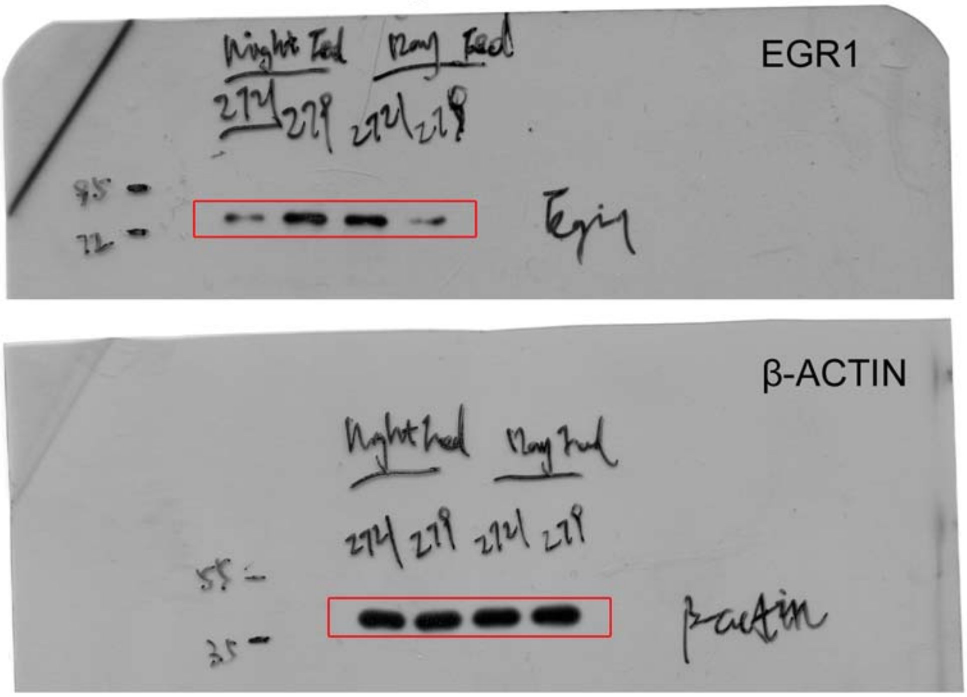

Supplementary Figure 9. Full-length images of the immunoblots in Figure 7b.

Red line boxes indicate the cropped images used in Figure 7b. β-ACTIN was used as an internal control.

Supplementary Table

Supplementary Table 1. PCR primers and siRNA sequences.

Gene	Forward/Reverse primer
Clock	5'-CACTCTCACAGCCCCACTGTAC-3'
	5'-CCCCACAAGCTACAGGAGCAGT-3'
Bmal1	5'-TGGAGGGACTCCAGACATTC-3'
	5'-TGGGACTACTTGATCCTTGG-3'
Rev-erbα	5'-TGCAGGCTGATTCTTCACACA-3'
	5'-AGCCCTCCAGAAGGGTAGGA-3'
Rev-erbβ	5'-CGCACATTGCCGATATAGGAGG-3'
	5'-GAGACTGCCACCACCACGTACT-3'
Rorα	5'-CCAACCGTGTCCATGGCAGAAC-3'
	5'-GCACACAGCTGCCACATCACCT-3'
Rorγ	5'-GGCAGCGCACCAACCTCTTTTC-3'
	5'-CTGGTCATTCTGGCAGAGCTCC-3'
Cry1	5'- AGCGCAGGTGTCGGTTATGAGC-3'
	5'- ATAGACGCAGCGGATGGTGTCG-3'
Cry2	5'-TGGGCATCAACCGATGGAG-3'
	5'-CCCATTCCTTGAACAGCCTTG-3'
Per1	5'-AACGGGATGTGTTTCGGGGTGC-3'
	5'-AGGACCTCCTCTGATTCGGCAG-3'
Per2	5'-TGATCGAGACGCCTGTGCTCGT-3'
	5'-CTCCACGGGTTGATGAAGCTGG-3'
Egr1	5'- GTCCTTTTCTGACATCGCTCTGA-3'
	5'-CGAGTCGTTTGGCTGGGATA-3'
36B4	5′-GAAACTGCTGCCTCACATCCG-3′
	5′-GCTGGCACAGTGACCTCACACG-3′
Scrb	5’-UUCUCCGAACGUGUCACGU-3’
	5’- ACGUGACACGUUCGGAGAA-3’
Egr1 siRNA	5′-UCUCCCAGGACAAUUGAAAUUUGCU-3′
	5′-AGCAAAUUUCAAUUGUCCUGGGAGA-3′
Per1 siRNA	5′-CCAGUACAACCAAGCGUAA-3′
	5′-UUACGCUUGGUUGUACUGG-3′
Per1 (ChIP)	5′-AGACATCCTGATCGCATTGGCTGA-3′
	5′-TGGGAGGCGGGTTGCATAAT-3′
Egr1 (ChIP)	5′-CTCCCTCACTGCGTCTAAGG-3′
	5′-CACCCAGAATCGAAAGGCTA-3′
